# Neurons in the Hippocampus of Crows Lack Responses to Non-spatial Abstract Categories

**DOI:** 10.3389/fnsys.2018.00033

**Published:** 2018-07-18

**Authors:** Helen M. Ditz, Jennifer K. Kupferman, Andreas Nieder

**Affiliations:** Department of Animal Physiology, Institute for Neurobiology, University of Tübingen, Tübingen, Germany

**Keywords:** crows, avian hippocampus, NCL, categories, numerosity

## Abstract

Lesion studies suggest a role of the avian hippocampus in spatial and episodic memory. However, whether the avian hippocampus is also involved in processing categorical information and non-spatial working memory contents remains unknown. To address this question, we trained two crows in a delayed-match-to-sample test to assess and briefly memorize the number of items in dot displays, i.e., their numerosity. We recorded neuronal activity in hippocampus while crows solved this task. Hardly any hippocampal neurons responded to the category ‘numerosity,’ during neither sample presentation, nor during the memory delay. This was in striking contrast to previous recordings in the telencephalic association area ‘nidopallium caudolaterale’ (NCL) of the same crows, in which we previously reported an abundance of numerosity-selective and working memory-selective neurons. Our data suggest that categorical information is not processed in the avian hippocampus.

## Introduction

Categorization is of adaptive value for many living organisms. It allows for adequate responses to its environment. The mammalian hippocampus is a key area for spatial categorization (O’Keefe and Dostrovsky, 1971), but is most likely not involved in non-spatial categorical processing: bilateral lesions restricted to the hippocampus in rats left the performance on a delayed non-matching to sample task unaltered compared to healthy controls (Mumby et al., 1992). Similar results were found in macaques (Alvarez et al., 1995; Murray and Mishkin, 1998). The role of the avian hippocampus in non-spatial tasks is still debated.

Ontogenetically, the avian and the mammalian hippocampus both originate from the same structure: the medial pallium (Székely, 1999; Atoji and Wild, 2006; Medina and Abellán, 2009; Allen and Fortin, 2013). However, differences in cytoarchitecture and neurochemistry between aves and mammals are present despite their shared origin (Rattenborg and Martinez-Gonzalez, 2011). Commonly, the avian hippocampus is defined as the pallial area medial to the paraventricular sulcus (Atoji and Wild, 2006). This definition includes medial parts of the parahippocampal area (Karten and Hodos, 1967), but in exchange also includes possible homologs of CA1, CA3, and the dentate gyrus. The dorsolateral hippocampus is proposed to be a homolog of the mammalian entorhinal cortex (Rattenborg and Martinez-Gonzalez, 2011), as it also functions as the main input structure to the hippocampus (Atoji and Wild, 2006). While some functional resemblances between the hippocampi of mammals and aves are revealed, there are still questions remaining.

If the avian hippocampus is involved in categorical processing, it should receive input from the highest cognitive center in the avian telencephalon, the nidopallium caudolaterale (NCL). Based on anatomical and physiological features, the NCL is considered to be the avian prefrontal cortex (PFC) analog (Veit and Nieder, 2013; Veit et al., 2014; Moll and Nieder, 2015; Güntürkün and Bugnyar, 2016; Ditz and Nieder, 2016a,b; Nieder, 2017). Recordings showed that NCL neurons are involved in a variety of executive processes, such as working memory (Veit et al., 2014), rules (Veit and Nieder, 2013), cross-modal associations (Moll and Nieder, 2015), and numerical competence (Ditz and Nieder, 2015; Wagener et al., 2018) processing of visually presented items in a set, i.e., numerosities. Neurons in the NCL are tuned to the shown quantity by increasing their firing rate to their preferred numerosity (Ditz and Nieder, 2015, 2016b). NCL neurons encode visual numerosities during its presentation, as well as show sustained activity during memorization of numerosities. It is unknown how information from the NCL is transferred to the hippocampus. No direct connection between the hippocampus and the NCL has been found (Leutgeb et al., 1996; Kröner and Güntürkün, 1999; Atoji et al., 2002). Some researchers suggest a connection from the dorsal ventricular ridge to the parahippocampal area and from there to the hippocampus (Allen and Fortin, 2013), but others failed in finding such a connection (Leutgeb et al., 1996; Székely and Krebs, 1996; Atoji et al., 2002). Another indirect route exists from the dorsal ventricular ridge via hyperpallium to the hippocampus (Rattenborg and Martinez-Gonzalez, 2011).

In this study, we tested whether neurons in the avian hippocampus contribute to visual non-spatial working memory. We recorded single-cell activity from the hippocampus of crows that performed a delayed match-to-sample task with numerosities. If the hippocampus is involved in categorization and working memory tasks, we expected to find categorical neurons during sample presentation as well as stimulus-specific working memory cells. We compared the hippocampus data to previously reported NCL recordings in the same task (Ditz and Nieder, 2015).

## Materials and Methods

### Subjects

One male and one female hand-raised carrion crow (*Corvus corone corone*) were trained to discriminate numerosities in a delayed match-to-numerosity task. Both crows originated from the institute’s own breeding facility. The crows were housed in social groups in indoor aviaries (Hoffmann et al., 2011). Crows were maintained on a controlled feeding protocol and earned food via rewards during and, if necessary, after training. Both crows participated in earlier studies on similar topics. All animal procedures were approved by the national authority (Regierungspräsidium Tübingen).

### Apparatus

The crows were placed in front of a touch screen (ART-Development PS-150, 15″, 60 Hz refresh rate) inside an operant conditioning chamber. Sitting on a wooden perch, the crows had a viewing distance to the screen of 14 cm. The crows had to maintain a stable head position throughout a trial. This was achieved by an infra-red light barrier and a foil attached to the crows head; only when the head was within the light barrier a trial would start. Once the test phase occurred, crows answered by leaving the light barrier with their heads. If a crow moved its head prematurely [i.e., before the test phase(s)], the trial was aborted and repeated at a later time point. Correctly completed trials yielded food reward (*Tenebrio molitor* larvae or bird seed pellets) accompanied by a sound. The reward was delivered via an automated feeder. Incorrect trials resulted in a light flash, a different sound, and a time-out. The stimuli were presented and behavioral data were stored with the program Cortex (National Institute of Mental Health).

### Behavioral Protocol

**FIGURE 1:**
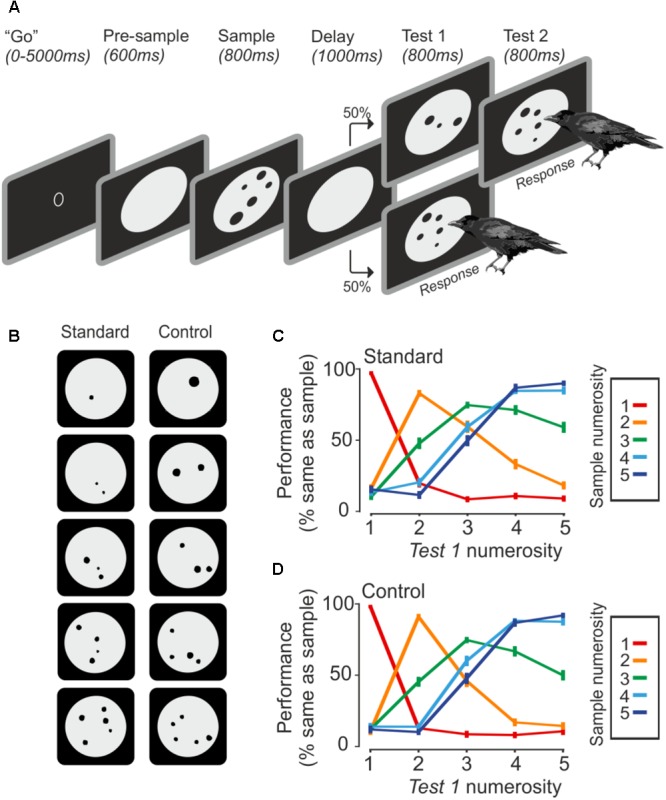
Task and behavior. **(A)** The crow initiates a trial by entering the light barrier when the ‘go’ stimulus is shown. Upon entering, the trial starts with a pre-sample phase, followed by the sample presentation. The crow has to remember the seen number of dots until a test stimulus comes up, which is either the same number of dots, which requires a response by the bird, or a different number of dots, which requires the bird to wait until the match appears. **(B)** Example stimuli. Control stimuli equate area and density over all set sizes. **(C,D)** Averaged behavior during standard and control trials (error bar ± SEM) over both birds and sessions (*n* = 75). Color indicates the sample numerosity; *X*-axis denotes the test numerosity. The *Y*-axis indicates the probability that a crow judges the sample and test numerosity as being equal.

The ability of crows to discriminate numerosities was tested with a delayed match-to-numerosity task (**Figure 1A**). A “go” stimulus indicated that the crow could initiate a new trial. A new trial started as soon as the crow entered the light barrier with its head. Upon entering the light barrier, a gray background circle appeared for 600 ms, followed by a sample dot display for 800 ms. The crow had to memorize the number of dots for 1000 ms during the delay period. Either the upcoming test phase showed the same number of dots as in the sample display (match), or the test contained a differing number of dots (non-match). Both occurrences were equally likely. The crows were trained to respond by leaving the light barrier whenever the number of dots in the sample and test displays were equal. If the first test stimulus was not equal to the sample, the crow had to wait 800 ms until a second test stimulus appeared which was always a match. Responses to non-matches or the omission of a response to a match were counted as error. The arrangement of dots in test and sample stimuli was always different.

### Stimuli

The presented number of dots varied from one to five items (**Figure 1B**). The dots (0.4° to 2.5° of visual angle) were randomly drawn within a gray background circle, but dots were never overlapping. The stimuli were created with a custom-written MATLAB script and exchanged daily. For each numerosity, 8 standard and 8 control stimuli were used, which totals 80 unique stimuli per session. The standard stimuli were unconstrained besides the restriction that dots must not overlap. Control stimuli all had an equal amount of cumulative surface area (equal area) and density was kept constant as well (equal density). Density was calculated by averaging the distance of each dots center to all other dot centers. Standard and control trials alternated randomly. The daily stimuli exchange paired with low-level visual controls for overall area and density across numerosities guaranteed that the crows used only the numerical features of the stimuli to solve the task.

### Surgery and Recordings

**FIGURE 2:**
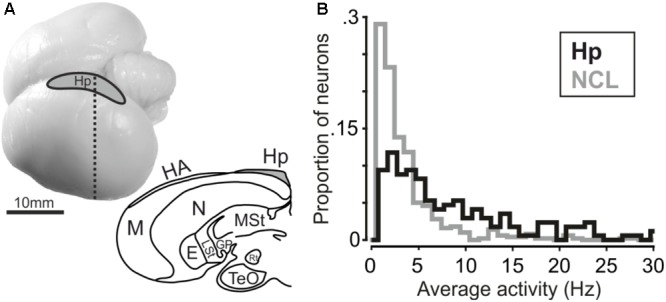
(**A**, top) Carrion crow brain with recording site. (**A**, bottom) Coronal section of a carrion crow brain; dashed line in the upper panel indicates section level. E: Entopallium; GP: Globus Pallidus; HA: Apical Hyperpallium; Hp: Hippocampus; LSt: Lateral Striatum; M: Mesopallium; MSt: Medial Striatum; N: Nidopallium; Rt: Nucleus Rotundus; TeO: Optical Tectum. **(B)** Histogram depicting average firing activity of hippocampus and NCL neurons. Bin size is 1 Hz.

Surgeries were conducted under general anesthesia (50 mg of ketamine with 5 mg of xylazine/kg body weight, i.m.). The head in the stereotaxic holder was placed at 0 (horizontal) beak bar position. The hippocampus can easily be delineated as lying next to the hemispheric midline and anterior to the cerebellum. The electrode-clusters were chronically implanted in the left hippocampus (**Figure 2A**) at AP 11–18 mm, ML 0–1 mm (directly next to the longitudinal fissure) for crow A (16 electrode cluster) and at AP 12–15 mm, ML 0–1 mm for crow J (8 electrode cluster). For recording, the electrodes were lowered vertical to the stereotaxic frame. The clusters were wired to a connector with amplifier. For both clusters, always four electrodes were attached to one microdrive, resulting in four microdrives for crow A and two for crow J. The reference-pin was inserted 1 cm anterior of the actual implant. Electrodes were glass-coated tungsten electrodes with 2MΩ impedance (Alpha Omega, Israel). Stereotactic coordinates were obtained from the jungle crow atlas (Izawa and Watanabe, 2007). After surgery, the crows received analgesics (Morphasol, 1 mg/kg, i.m.). Head posts to hold the reflector for the light barrier were implanted earlier.

Before each recording session, the electrodes were manually lowered further into the tissue until a good neuronal signal at least one electrode per microdrive was detected. Electrodes were retracted once the depth of 2 mm was reached. Then, after 2–3 days of rest, the electrodes were step wise lowered again for recording. Neuronal signal amplification, filtering, and spike waveform digitization were conducted with the Plexon system. For recording, the connector on the crows’ head was connected via a cable to a second Plexon amplifier and filter outside the setup. Spikes were sorted manually using the Plexon Offline Sorter (version 2.8.8).

### Data Analysis

All data analysis was conducted with MATLAB (MathWorks, R2016a). Behavioral performance (% same as sample) was calculated by dividing the number of trials when the bird answered to the first test stimulus for one condition by the total number of trials for the respective condition. Behavioral performance curves were calculated for each session separately for standard and control trials.

Neural data analysis included all neurons that were recorded for at least three repetitions of each sample numerosity and stimulus type (standard and control) and had an average firing rate > 0.5 Hz over the course of a trial.

Sample-responsive neurons were determined by comparing spiking activity during baseline – defined as pre-sample phase (offset 100 ms, duration 500 ms) – and the sample phase (offset 100 ms, duration 800 ms). Delay-responsive neurons were determined similarly, except that baseline activity was compared to the spiking activity during the delay phase (offset 200 ms, duration 900 ms). Numerosity selectivity was calculated separately for the sample and delay phase with a two-factorial ANOVA (numerosity and stimulus type). The ANOVA selects for any neuron that exhibits a significantly different firing rate to at least one of the shown numerosities compared to the other numerosities. Sample activity was measured in an 800 ms window shifted by 100 ms from stimulus onset. Delay activity was taken over a 900-ms time-window shifted by 200 ms from delay onset. Only neurons which were significant for numerosity (*p* < 0.01) without stimulus type effects nor interaction were categorized as numerosity selective.

To quantify the effect of numerosity, stimulus type, and their interactions on the population firing rates in percent-explained variance (PEV) analysis, we calculated the PEV using ω^2^. This measure reflects the amount of variance in the firing rates that is explained by the factors. ω^2^ is defined as

$$\begin{array}{c} \omega^{2}=\frac{SS_{group}-[{\left(df-1\right)}^{*}MSE]}{SS_{total}+MSE} \end{array}$$

with SS meaning the sum of squares, *df* the degree of freedom, and MSE the mean squared error. The terms were calculated using a two-way ANOVA with a 300-ms sliding window, advanced in steps of 20 ms.

For the state space analysis, we performed a principal component analysis (PCA) which offers a dimensionality reduction of the population activity and was performed to capture task relevant information that is not represented by individual neurons, but rather on a population level (Cunningham and Yu, 2014).

For this analysis, the neuronal data for each neuron was averaged for each sample numerosity, smoothed by a 100-ms Gaussian kernel, binned in 50 ms steps, and then *z*-scored before performing the PCA (detailed description in Ott and Nieder, 2016). The resulting state space trajectories over the first three most meaningful principal components (PC) represent the firing rate changes in the population due to task manipulations over time. In the hippocampus, the first three PCs captured 22.7% of the firing rate variance; in the NCL, they captured 34.4%.

We evaluated the populations processing of numerical information by calculating the Euclidean distances between population trajectories for all numerosity combination using all PCs (*n* = 170 in hippocampus; *n* = 501 in NCL). For clarity, Euclidean distance trajectories with equal numerical distances were pooled. For example, the distance-trajectory for distance 3 was pooled over the distance-trajectories from 1v4 to 2v5.

## Results

Two crows performed a delayed match-to-numerosity task with up to five items as sample and test stimuli (**Figure 1A**). The crows saw a sample numerosity, which they had to assess and memorize for 1 s. If the upcoming test stimulus contained the same number of dots, the crows had to respond by moving their heads, thus leaving the light barrier. If the test stimulus differed in the number of dots, the crows had to wait until a match appeared (see Ditz and Nieder, 2015). Daily stimuli exchange and control of co-varying visual parameters ensured an exclusive use of numerical information to solve the task.

### Behavioral Performance

Both crows were proficient in the task (crow A: 76.7 ± 3.8%, *n* = 43 sessions; crow J: 77.2 ± 4.2%, *n* = 32 sessions). The crows completed an average of 500 ± 77 trials per session (correct and incorrect trials). Both crows readily generalized to the control stimuli. Performance for both stimuli sets was similar (**Figures 1C,D**). The similar performance to the baseline and generalization stimuli indicates an exclusive use of numerical information. For both stimulus sets, crows made fewer errors when sample and test stimuli were numerically further apart (numerical distance effect) and when the total magnitude was smaller (numerical magnitude effect; 1v2 generated fewer errors than 4v5, albeit the numerical distance is equal).

### Neuronal Results

We recorded 170 single units in the left hippocampus of two crows (crow A: 105, crow J: 65 neurons). Hippocampal results are compared to neuronal data recorded in the (left) NCL (*n* = 501 neurons) in the same birds with the same task (data from Ditz and Nieder, 2015). Average firing activity was significantly higher in hippocampal neurons compared to neurons in the NCL (Mann–Whitney *U*-test, *n*_Hp_= 170, *n*_NCL_= 501, rank-sum: 147247, *p* < 0.001, **Figure 2B**). Median activity amounted to 5.9 Hz in hippocampus and 2.4 Hz in NCL. Next, we tested whether neurons in the hippocampus and NCL modulated their firing rate during the course of a trial in response to the shown numerosity.

**FIGURE 3:**
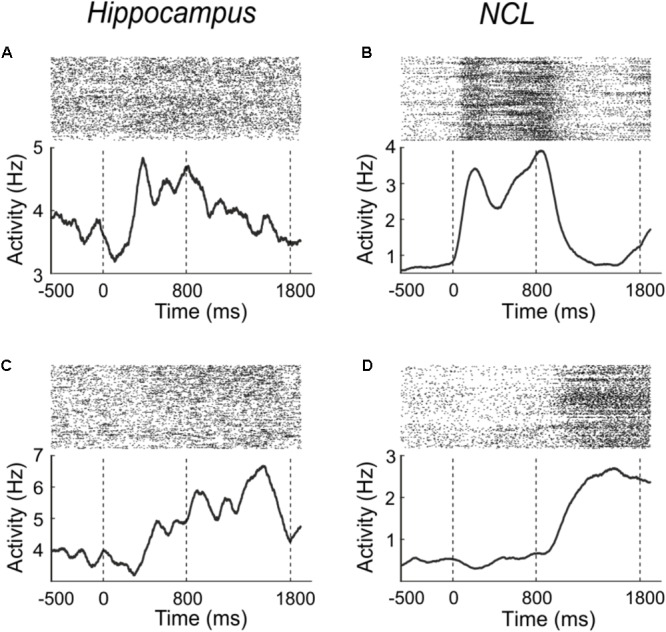
Example neurons from the hippocampus **(A,C)** and NCL **(B,D)**, which modulate their firing rate in response to the sample **(A,B)** or delay **(C,D)** phase. Sample onset is indicated by a dashed line at 0 ms; delay onset at 800 ms. Upper panels: dot raster histogram; one row is one trial, one dot is one action potential. Lower panels: averaged spike density function smoothed with a 150-ms Gaussian kernel.

To determine how many neurons were generally responsive to the sample-phase, we compared firing rates from the pre-sample period with the sample period. Example neurons which increase their firing rate in response to the stimulus are shown in **Figures 3A,B** for both brain areas. **Figure 3A** shows a hippocampus neuron, which increases its firing rate in response to sample presentation; an equivalent example neuron from the NCL is shown in **Figure 3B**.

In the hippocampus 24.1% (41/170) of the randomly sampled population modulated their firing rate in response to the sample (Wilcoxon test, *p* < 0.01). The neurons response properties did not seem to change across recording depth nor anterior-posterior coordinates. In the NCL, twice as many neurons (49.5%; 248/501) of the population responded to the sample presentation (Wilcoxon test, *p* < 0.01). The proportion of neurons in hippocampus and NCL that changed their firing rate in response to the sample phase differed significantly between the brain regions (χ^2^-test, χ^2^ = 33.35, *p* < 0.001).

Example neurons which significantly (Wilcoxon test, *p* < 0.01) changed their activity during the delay phase in comparison to the pre-sample phase are shown in **Figures 3C,D**. **Figure 3C** shows a hippocampus neuron which increases its firing rate in response to the delay; an equivalent example neuron from the NCL is shown in **Figure 3D**.

In the hippocampus, 27.1% (46/170) of the population changed their firing rate in the delay phase compared to the pre-sample phase (Wilcoxon test, *p* < 0.01). In the NCL, proportionally twice as many neurons (53.3%, 267/501) modulated their firing rate in response to the delay (Wilcoxon test, *p* < 0.01). The proportion of neurons in hippocampus and NCL differed significantly between the brain regions (χ^2^-test, χ^2^ = 35.1, *p* < 0.001).

### Processing of Numerosity Information

**FIGURE 4:**
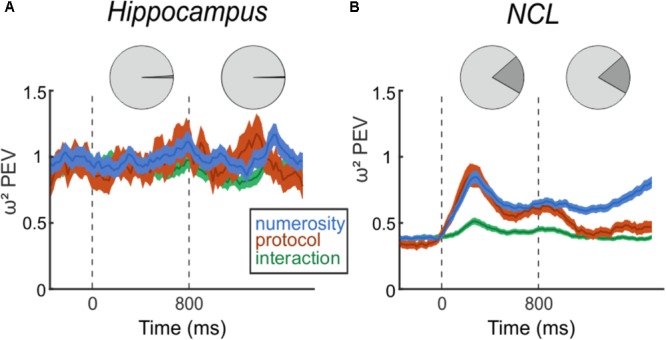
Effect size of numerosity stimuli on the firing rates. **Top**: Pie charts indicating the percent of numerosity selective neurons in the trial phases. **Bottom**: Time-course of percent-explained variance (PEV ω^2^) by the factors *number, protocol*, and *interaction* separately for hippocampus **(A)** and NCL **(B)**, over the entire population of recorded neurons. Dashed lines indicate sample (0 ms) and delay (800 ms) onset. Shading: ± SEM.

Next, we assessed whether neurons in the hippocampus respond to the number of items in a set, i.e., numerosity. This was first assessed with a two-factorial ANOVA (*p* < 0.01) with numerosity and protocol as factors. We found two (1.2%, 2/170) neurons that responded to numerosity in the sample phase and one neuron (0.6%, 1/170) that was numerosity selective in the delay (**Figure 4A**, top). With a significance threshold of 1% we assume the number of neurons we found to be at chance level. In the NCL, however, we found 99 (19.8%, 99/501) neurons responding to numerosity in the sample phase and equally as many in the delay phase (19.8%, 99/501), including only neurons that solely respond to numerosity and not to protocol nor interactions (**Figure 4B**, top). Of the 99 cells, which are numerosity selective in the delay phase, 30 are numerosity selective in the sample phase, too.

Next, we analyzed the entire population of neurons, irrespective of ANOVA-selectivity, but separately for both brain regions. We calculated the PEV (ω^2^) to quantify the effect of the task manipulations on the firing rates over the course of a trial (**Figure 4**). In the hippocampus, the PEV in the sample and delay period is unchanged compared to the presample period (**Figure 4A**). This consistency indicates that observed firing rate changes between presample and sample, or presample and delay phase are not due to processing of numerical information. In contrast to the hippocampus, the PEV in the NCL (**Figure 4B**) drastically increases with sample onset and numerosity information persists throughout the delay and ramps up toward the test phase where the information is needed.

**FIGURE 5:**
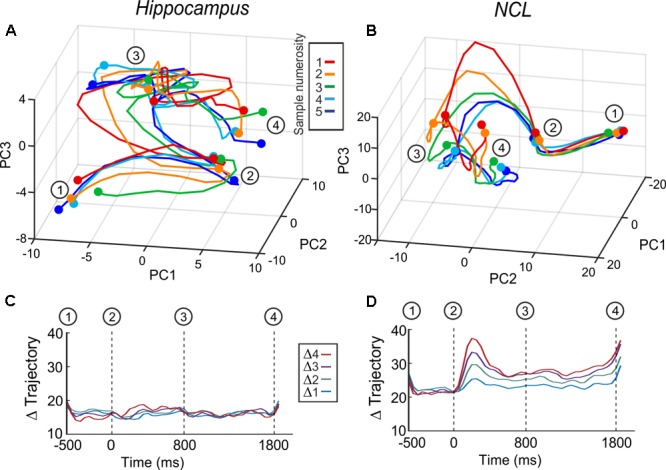
Averaged whole-population state-space trajectories in hippocampus **(A)** and NCL **(B)** separated by sample numerosity. **(C,D)** Inter-trajectory Euclidean distances between the trajectories of the different sample numerosities over all principle components. **(C)** Hippocampus and **(D)** NCL. Δ trajectories with the same distance are grouped and averaged (e.g., Δ2 is the average of the inter-trajectory distances of the distances of 1v3, 2v4, 3v5).

To verify the indication that hippocampus is not involved in numerosity processing, we applied another population analysis: a state space analysis. This analysis is sensible to a possible coordination of responses across neurons, which would not show at the level of single neurons (Cunningham and Yu, 2014). Population responses are represented in state space by the first three principal components (**Figures 5A,B**). The trajectories represent population firing rates over time in response to numerosity. An increase in distance between the trajectories – once numerical information is presented – implies that the population discriminates between numerosities. We calculated all inter-trajectory distances using all PCs; the resulting inter-trajectory distances are constant over all trial phases for the hippocampus (**Figure 5C**), whereas in the NCL the distances increase once the sample is presented and continue to be above the level of the presample phase throughout the delay (**Figure 5D**). The state space analysis further supports the result that the avian hippocampus is neither involved in the encoding nor in the retention of numerical information.

## Discussion

We recorded neuronal activity in the hippocampus of crows that performed a delayed match-to-numerosity task. Approximately, a quarter of the sampled hippocampus cells modulated their firing rate in response to visual stimuli during sample presentation and another quarter during the delay. Hardly, any hippocampal cells responded to the category of the stimulus, i.e., numerosity, in either of the phases. The results in the hippocampus are in stark contrast to recordings from the corvid NCL, in which a substantial proportion of the cells responded to category during both encoding and working memory phases. This discrepancy in category encoding neurons begs the question whether or not the avian hippocampus is involved in non-spatial working-memory tasks.

### Lack of Visual Categorical Cells in the Avian Hippocampus

In our study, only a quarter of the cells in the hippocampus were visually driven, which is a lower proportion compared to previous findings recorded in pigeon hippocampus (46%, 31/67; from Scarf et al., 2016). In the latter study, pigeons repeatedly observed 12 stimuli and had to peck within 2 s upon stimulus presentation. The stimuli entailed pictures of another pigeon, colors (red and green), real world items, or simple black patterns. Importantly, however, neurons that fired stimulus specific were also scarce in these pigeons (1/67); the one stimulus-specific neuron they found responded to a green circle (Scarf et al., 2016).

We conclude that the avian hippocampus is not involved in encoding categories, even though there is an abundance of categorical cells in the NCL, which has an indirect connection to the hippocampus (Rattenborg and Martinez-Gonzalez, 2011). The NCL is involved in many cognitive control functions, such as categorizing (Ditz and Nieder, 2015, 2016b), rule and association learning (Veit and Nieder, 2013; Moll and Nieder, 2015, 2017; Veit et al., 2015b), and also spatial representations (Veit et al., 2015a). Spatial representations were tested in crows that had to memorize a sample picture for 1 s. Then, in the test phase, the crow had to choose which of the four present images it previously saw. One-third (36%, 83/231; from Veit et al., 2015a) of the neurons in the NCL encoded the position of the image, independent of image identity. We conclude that the NCL processes categorical and spatial information. However, this categorical information in NCL does not stem from nor is it passed on to the hippocampus. The indirect connection from NCL to hippocampus might channel spatial information from the NCL to the hippocampus.

### Lack of Categorical Working Memory Cells in Hippocampus

While a quarter of the hippocampal cells modulated their firing rate during the working memory phase, we found only one stimulus-specific hippocampal working memory neuron. An experiment by Sahgal (1984) with pigeons implies that the hippocampus might be involved in working memory. Pigeons performed a pair-comparison task by indicating the same- or differentness of an image to a previous image by either pecking to the right or left side of the image. Bilateral hippocampal lesions impaired performance on this task. While Sahgal (1984) concluded that the impairment must originate from an impaired visual working memory, it is also likely that the drop in performance resulted from an impaired spatial memory (Colombo and Broadbent, 2000). Subsequent experiments by other researchers eliminated the spatial component of the task by utilizing a delayed match-to-sample task with colors. Pigeons, chickadees, and juncos with bilateral hippocampus lesions were unaffected in their performance (Good and Macphail, 1994; Hampton and Shettleworth, 1996; Colombo et al., 1997b), indicating that the earlier observed deficit by Sahgal (1984) stemmed from impaired spatial memory rather than impaired working memory (Colombo and Broadbent, 2000). The lack of stimulus-specific working memory cells in our experiment also suggests that the avian hippocampus is negligible for (non-spatial) working memory.

In contrast to the hippocampus, the NCL does contain a substantial proportion of working memory neurons. Abstract categories such as numerosity (Ditz and Nieder, 2016b), but also simple pictures – including pictures of faces, flowers, and animals (Veit et al., 2014) – are represented and maintained by sustained activity over a period of 1 s. Moreover, the sustained responses during working memory of neurons in the crow NCL are predictable of performance in a trial (Veit et al., 2014; Ditz and Nieder, 2016b).

### Comparison of the Avian and Mammalian Hippocampus

The avian and the non-human mammalian hippocampus share many similarities. Both are involved in spatial memory (Colombo et al., 1997a; Bingman et al., 2005) and episodic memory (Clayton and Dickinson, 1998; Salwiczek et al., 2010). Spatial memory was investigated by exploiting the natural navigational abilities of homing pigeons. If young pigeons were lesioned in the hippocampus, their ability to navigate home was impaired (Bingman et al., 2005). Episodic memory was investigated via food hoarding bird species. Food hoarding is a process where food is cached for later retrieval. Thereby, the animal must remember what food was hidden where and at which time-point for successful retrieval later on (Clayton et al., 2003; Zinkivskay et al., 2009; Feeney et al., 2011). Hoarding is a memory-intensive task. It should therefore lead to an increased hippocampus volume in hoarding species. This was indeed the case in closely related hoarding versus less/non-hoarding species (Clayton, 1995; Basil et al., 1996; Volman et al., 1997). Inactivation of the hippocampus in black-capped chickadees demonstrated an impairment in retrieving caches (Shiflett et al., 2003). Another similarity is the apparent non-involvement in delayed match-to-sample (or non-match-to-sample) tasks. Bilateral lesions confined to the hippocampus of macaques and rats had no effect on the performance in such tasks (Mumby et al., 1992; Alvarez et al., 1995; Murray and Mishkin, 1998).

The lack of categorical cells in the avian and the (non-human) mammalian hippocampus is in strong contrast to the human hippocampus, given the abundance of categorical cells there (12% categorical cells in Kamiñski et al., 2017; 15% in Kornblith et al., 2017). In the human studies, categorical cells were determined via a (modified) Sternberg task: the participants viewed up to four images with delays between the stimuli. After image presentation, two test images were shown simultaneously. The participants had to indicate which of the two images they had previously seen (Kamiñski et al., 2017; Kornblith et al., 2017). Neurons in the human hippocampus were selective to categories such as faces, objects, gender, facial expressions, person identity, and landmarks (Fried et al., 1997; Kreiman et al., 2000; Quian Quiroga et al., 2005; Kamiñski et al., 2017; Kornblith et al., 2017). Notably, coding for numerical categories was not tested in humans, yet. Hence, the reasons for the discrepancy in categorical cells could be that the presented stimuli in our experiment were not diverse enough. The stimuli in a pigeon study (Scarf et al., 2016) were more diverse, but the content of the stimuli had no behavioral relevance to the birds, which might influence results as well.

Working memory cells in the human hippocampus were recently found; these neurons modulated their activity during sample presentation as well as in the working memory phase specific to the item held in memory (Kamiñski et al., 2017; Kornblith et al., 2017). A subset of sample selective cells was also active during the delay; the delay cells were not a different population of neurons. The persistent activity of these neurons was a predictor of performance in a given trial (Kamiñski et al., 2017).

## Conclusion

Our data suggest that the avian hippocampus is not involved in categorical processing. This is in line with previous bird studies (Good and Macphail, 1994; Hampton and Shettleworth, 1996; Colombo et al., 1997b; Scarf et al., 2016). Furthermore, the results add to the line of evidence that the avian and (non-human) mammalian hippocampus are largely functional homologs. Anatomically, both hippocampi share many connections – such as the input from the contralateral hippocampus, thalamus, bidirectional connections with hypothalamus and diagonal band, and outputs to septal nuclei (Colombo and Broadbent, 2000) – but there are differences in connection as well. Specifically, the mammalian hippocampus interacts with many parts of the neocortex, whereas the avian hippocampus shares no direct connection with pallial association areas (Rattenborg and Martinez-Gonzalez, 2011). The implications of the lack of connections to pallial association areas still need to be investigated.

## Ethics Statement

This study was carried out in accordance with the recommendations of local ethics committee. The protocol was approved by the Regierungspräsidium Tübingen.

## Author Contributions

HD, JK, and AN designed the experiments. HD and JK conducted the experiments. HD analyzed the data. HD and AN wrote the paper.

## Conflict of Interest Statement

The authors declare that the research was conducted in the absence of any commercial or financial relationships that could be construed as a potential conflict of interest.
